# The Growth of Therapeutic Nihilism

**Published:** 1911-01

**Authors:** Frederic S. Mason

**Affiliations:** New York


### The Growth of Therapeutic Nihilism

By FREDERIC S. MASON, B.Sc., Ph.G., M.D.
New York

[From *Monthly Cyclopedia and Medical Bulletin*, July, 1910]

WHEN in Europe last year, it was somewhat of a surprise to me to find that loss of confidence in medication is gaining ground on the Continent as in America. In conversation with young physicians in France, Germany and Switzerland, this pessimism was painfully marked in contrast with the optimistic views entertained on surgery and pathology. The older practitioners do not seem to go outside the officinal and semi-officinal remedies consecrated by usage, and are perhaps better therapeutists because they have learned to appreciate some of these old drugs in their long career, while the younger members of the profession, with rare exceptions, regard almost every drug as a mere placebo. Mercury in syphilis, quinine in malaria, morphine and coal-tar derivatives for pain, are still used, it is true, but few others are recognised as of real value. This may be accounted for, in a measure, by the surfeit of new therapeutic agents which chemists have brought forward in recent years. Yet patients do not appreciate expectant treatment, and look to their medical adviser not only for a correct diagnosis, for lessons on hygiene, diet and the like, but also for relief of symptoms by medication.

As Sajous pointed out recently in one of his admirable communications, the non-progressive position of present-day therapeutics must be attributed largely to the want of a more thorough knowledge of the true action of drugs, few of which have been scientifically tested from a physiologic standpoint, although an immense number of unclassified observations exist; and until this knowledge is accurately defined and laws established, no real advance can be expected.

It is not disputed that therapeutics is encumbered with rank empiricism; many of the so-called "classical" remedies have no true scientific value, and the number of those recognised could with advantage be considerably reduced. Even the most rabid therapeutic nihilist, however, will not deny that certain drugs do modify physiologic functions, and would gladly use them if their employment were reduced to a positive science, that is, if the accumulated facts had been classified and a reliable basis of scientific treatment could be established.

Now that physical diagnosis, *x*-ray pictures, laboratory examination of urine, stomach-contents, feces, sputum, blood, and the like, are recognised duties of the medical attendant, and that he is instructed in the methods of such examinations, he expects to find reliable data with regard to drugs and their uses. The study of therapeutics is so complicated that the average practitioner finds it impossible to attempt for himself the investigation of more than a very few drugs; hence he soon gets into a rut, and it is rare to find even a prominent man prescribing a varied list of officinal or unofficinal remedies. We see, then, how essential it is for the rehabilitation of therapeutics that specialists should thoroughly revise by investigation the claims made for even our most important remedies.

In order to do this, a qualified body of independent workers should undertake to verify the reputed properties of the officinal and more modern non-officinal remedies. The services of chemists and pharmacists will also be necessary, but they will be less important than those of biologists, physiologists, pathologists and clinicians; for chemistry belongs to the most exact sciences, and our knowledge of the actual composition of drugs and the best methods of compounding them have made remarkable strides in comparison with that of drug action. Not only must experiments be made on normal human beings, but also on animals. Until now, the action of drugs has best been known in its application to pathologic subjects, but we should know also their influence on normal subjects, in order to verify their varied effects when the many factors inherent to disease are present.

In this connection we would urge that this body of investigators should look for other means of administering drugs than by the mouth, which of all methods is the most undesirable, and has only its facility to recommend it.

Many therapeutic agents are powerful enough to be modified by or to modify the gastrointestinal secretions and often injuriously affect the mucosa and interfere with secretion. Investigators might, therefore, among other means, consider the advantages of introducing small doses of active drugs through the skin, not only intramuscularly, or by subcutaneous injection, but by inunction. Great progress on these lines is possible, for the skin can be made to absorb therapeutic agents rapidly by new methods which might be brought to the attention of the profession. It is doubtful whether the Council of Pharmacy of the American Medical Association has the facilities to undertake such work, and while the council has made a laudable attempt to purify the pharmaceutical atmosphere, the present campaign will appear childish in years to come, for it is a poor compliment to our modern colleges to believe that well-trained graduates will be likely to prescribe the unscientific preparations exposed in the *Journal of the American Medical Association.* Ardent therapeutists have perhaps been somewhat intimidated from fear of being accused of being influenced by manufacturing interests, so that hardly any physician of note would dare to publish favorable investigations concerning the exact physiologic action of a new drug. Progress on these lines, therefore, has hitherto come from Europe, more especially from Germany, where even the most eminent professors are not only willing but anxious to make such investigations.

The so-called "proprietary interests," however, certainly required restraint and the council's work was necessary before anything else could be attempted. Optimism in every branch of medicine is in the ascendency; I believe a reaction must surely come, that the old science of therapeutics will soon find earnest workers to take up investigations into the value of drugs in disease, and that finally more definite data will be obtained which will reinstate this now neglected branch of medicine in the prominent position which it once held.

